# Multi-Classification of Breast Cancer Lesions in Histopathological Images Using DEEP_Pachi: Multiple Self-Attention Head

**DOI:** 10.3390/diagnostics12051152

**Published:** 2022-05-05

**Authors:** Chiagoziem C. Ukwuoma, Md Altab Hossain, Jehoiada K. Jackson, Grace U. Nneji, Happy N. Monday, Zhiguang Qin

**Affiliations:** 1 School of Information and Software Engineering, University of Electronic Science and Technology of China, Chengdu 610054, China; kofijackson@uestc.edu.cn (J.K.J.); ugochinneji@std.uestc.edu.cn (G.U.N.); 2 School of Management and Economics, University of Electronic Science and Technology of China, Chengdu 610054, China; altabbd@uestc.edu.cn; 3 School of Computer Science and Engineering, University of Electronic Science and Technology of China, Chengdu 610054, China; mh.nkanta@std.uestc.edu.cn

**Keywords:** histopathological images, breast cancer, medical images, transfer learning, multi-head self-attention, image classification

## Abstract

Introduction and Background: Despite fast developments in the medical field, histological diagnosis is still regarded as the benchmark in cancer diagnosis. However, the input image feature extraction that is used to determine the severity of cancer at various magnifications is harrowing since manual procedures are biased, time consuming, labor intensive, and error-prone. Current state-of-the-art deep learning approaches for breast histopathology image classification take features from entire images (generic features). Thus, they are likely to overlook the essential image features for the unnecessary features, resulting in an incorrect diagnosis of breast histopathology imaging and leading to mortality. Methods: This discrepancy prompted us to develop DEEP_Pachi for classifying breast histopathology images at various magnifications. The suggested DEEP_Pachi collects global and regional features that are essential for effective breast histopathology image classification. The proposed model backbone is an ensemble of DenseNet201 and VGG16 architecture. The ensemble model extracts global features (generic image information), whereas DEEP_Pachi extracts spatial information (regions of interest). Statistically, the evaluation of the proposed model was performed on publicly available dataset: BreakHis and ICIAR 2018 Challenge datasets. Results: A detailed evaluation of the proposed model’s accuracy, sensitivity, precision, specificity, and f1-score metrics revealed the usefulness of the backbone model and the DEEP_Pachi model for image classifying. The suggested technique outperformed state-of-the-art classifiers, achieving an accuracy of 1.0 for the benign class and 0.99 for the malignant class in all magnifications of BreakHis datasets and an accuracy of 1.0 on the ICIAR 2018 Challenge dataset. Conclusions: The acquired findings were significantly resilient and proved helpful for the suggested system to assist experts at big medical institutions, resulting in early breast cancer diagnosis and a reduction in the death rate.

## 1. Introduction

Cancer is among the majority of deadly diseases, claiming the lives of millions of people each year. Breast Cancer (BC) is the most common cancer and the leading cause of death among women [1]. As per World Health Organization (WHO) data, 460,000 people die annually from BC out of 1,350,000 cases [2]. The United States (US) alone recorded about 268,600 instances of BC in 2019, setting a new record [3,4]. BC develops due to aberrant cell proliferation inside the breast [5]. The breast anatomy comprises several blood arteries, tendons and ligaments, milk ducts, lacrimal gland, and lymph ducts [6]. Benign carcinoma is squamous cell carcinoma that forms due to minor anomalies in the breast. Malignant carcinoma, in contrast, is classed as melanoma and further characterized as invasive carcinoma or in situ carcinoma [7]. Invasive BC expands to nearby organs and causes difficulties [8,9], whereas in situ carcinoma stays limited to its territory and does not affect surrounding tissues. To avoid future progression and problems, BC must be identified earlier and correctly classified as benign or malignant carcinoma. As a result, a prompt and accurate therapy may be devised, lowering the disease’s fatality rate. Diverse imaging techniques are used to identify BC, such as Histopathology (HP) [10], Computed Tomography (CT) [11], Magnetic Resonance Imaging (MRI) [12], Ultrasound (US) [13], Mammograms (MGs) [14], and Positron Emission Tomography (PET). Statistics reported in recently published studies on imaging methods [15] reveal that 50% of datasets utilized in BC-related research are MGs, 20% are US, 18% are MRI and 8% are HP. The remaining percentage includes commercial records and data from different forms [6,12,16]. Further studies prove that HP images do not offer binary identification and classifications but support the multiclass identification and classification of BC subtypes [17,18,19]. In this paper, a BHI dataset at various magnifications (40×, 100×, 200×, 400×) is studied. The preprocessing of various magnification varies. For instance, with 100× magnification, a specialist examines squamous development, mesenchymal involvement, and tumor localization to determine the carcinoma. Nevertheless, developing an accurate and fast model to evaluate BHI at various magnifications is difficult due to multiple factors such as variable pixel intensity, microscopic size nucleus, diverse image characteristics, a wide variation of nuclei, the existence of distortions, and so on. The current effort aims to create a deep learning-based attention model to categorize BHIs in various magnifications.

Several strategies have been studied for classifying BHIs under 100× magnification [20,21]. Conventional approaches are always focused on feature extraction. On the other hand, finding relevant handmade characteristics necessitates experience and expertise but these might fail to grasp all permutations in the dataset. Deep learning-based approaches have recently gained prominence as processing computing capacity has improved. Their ability to analyze end-to-end provides it a better choice for BHI classification. Convolutional layers are used in deep learning algorithms to extract input image features. These convolutional layers often extract unwanted features alongside the needed parts or overlook the essential features. However, the extracted features influence the result and choice of malignancy; thus, disregarding these aspects may result in incorrect image evaluation. As a result, the extracted characteristics by the convolutional layers of CNN are insufficient for classifying BHIs. We present an attention-based deep learning framework that employs global and local features to determine tumor malignancy. The mechanism of the human brain to interpret visual data while still analyzing the significance of input elements is known as attention. This neurological mechanism enables exclusive focus on a single piece of information while ignoring other discernible details. Nevertheless, in opposition to the competency of attention, the conventional and commonly used CNN classifier examines characteristics more broadly. It is not assured of extracting relevant clinical knowledge subconsciously comparable to trained networks [22]. Self-attention is a significant advancement of computer vision [23,24,25,26,27,28]. These advancements focus exclusively on essential features in an informal m with no external guidance. The CNN models serve as the backbone of the self-attention models. They are trained end-to-end, with no modifications in the training phase. Thus, employing self-attention processes inside conventional CNN yields several advantages in accuracy, comprehensibility, and robustness on clinical vision tasks.

### 1.1. Diagnostic Medical Methods Used in the Investigation of BC

Having mentioned several medical imaging methods used in diagnosing BC, this paper describes the imaging methods related to our task and why we chose histopathological images in this section. PET is an accepted imaging method that might provide handy information regarding BC; nonetheless, it is usually utilized for early grading of advanced or metastatic invasive and reactive breast cancers, assessing progress to therapies and detecting and localizing family history of the disease [29]. As a result, we did not include it in our discussion.

The most often and extensively used technique is MGs [30,31,32], as they are easily accessible as public datasets. MGs are small breast X-rays [33] that are simple and frequently employed as the initial test for BC identification [34]. Regrettably, because of the vast discrepancies in shape, the surface area of breast tissues and morphological form, these are not reliable as they are associated with health effects, including radiation exposure risks for carriers and radiologists and overdose of radiation effects for carriers [35]. Moreover, due to inadequate specificity, these techniques subject a considerable proportion of the population (65–85%) to unnecessary biopsy procedures [36,37]. Such unnecessary biopsies increase the hospitalization cost for individuals and cause mental stress. Due to such limitations, US imaging is considered a much better option for breast cancer diagnosis and detection [38,39].

US imaging can significantly boost detection accuracy by 17% while decreasing overall needless biopsy procedures by 40% [39] compared with MGs. Sonograms are another title for breast US in clinical medicine. US might be a superior option to MGs for BC assessment and diagnosis due to its adaptability, reliability, sensitivity, and selectivity [40]. On the other hand, BC lesion identification and classification with US imaging need radiologists’ experience and knowledge due to its complexity and speckle [41]. Aside from the complicated imaging form, US image-based assessment in female patients produces unsatisfactory false detection results and misclassification [42]. As a result, there is insufficient evidence to recommend the use of US in the diagnosis and treatment of BC.

MRI breast images yield better sensitivity for detecting BC in dense tissue [43]. MRI images provide a more thorough overview of breast tendons than CT, US, or MGs images because multiple samples from different angles constitute a patient’s breast image sample [44]. Since MRI scans are more comprehensive than other alternative imaging techniques, they may uncover tumors not apparent on different imaging techniques or be deemed malignant [45]. Despite MRI’s high sensitivity [46], its adoption for BC diagnosis is limited due to its expensive cost [47]. Conversely, newer MRI methods, such as DWI (Diffusion-Weighted Imaging) and UFMRI (Ultrafast Breast MRI), provide much improved diagnostic precision with faster processing efficiency and lower expenses [48,49].

HP is the process of removing a heap from a questionable anatomical and physiological spot for screening and extensive investigation by specialists [50]. In clinical medicine, this procedure is commonly referred to as a biopsy. Biopsy specimens are mounted over a microscope slide clouded with Hematoxylin and Eosin (H&E) for examination [51]. HP images come in two types: (i) Whole Slide Images (WS), which are computerized color imaging, and (ii) image patches derived from WSI. Several researchers have effectively employed HP images in the multiclassification of BC due to tissue level examination [17,18,19]. BC identification and classification with HP images has several benefits over MGs and other imaging alternatives such as MRI and US. In particular, HP images do not offer only binary identification and classifications but support multiclass identification and classification of BC subtypes. Table 1 illustrates the summary of the discussed Breast cancer modalities, its robustness, constraints and available datasets.

### 1.2. Related Studies

The AI approach’s classification of BHI has received much attention in the research field [10,65,66,67]. There are significant obstacles in developing AI systems to examine these images, such as cancerous specimen variability, illumination variations and hue variations, intraclass fluctuations, different magnifications, and the existence of abnormalities, among others. Researchers used the traditional technique and deep learning models, which are further explored below and summarized in Table 2.

Various conventional approaches to image analysis have been presented by numerous scholars [68,69,70,71]. These approaches include several phases, such as the preprocessing phase, region of interest segmentation phase, the extraction of features phase, and identification phase. In Refs. [71,72], Local Binary Patterns (LBP) were used for BHI categorization, while the authors of Ref. [73] used the frequency distribution index, in conjunction with contours, to identify meiosis. Unfortunately, due to the varied properties of cancerous images, appearance alone will be inadequate for effective image classification. Furthermore, support vector machines (SVM) [71] and decision trees (DT) [74,75] have been widely investigated for image classification. These strategies focused on data preprocessing since it significantly influenced the recognition rate. Such techniques depend on characteristics that have been handcrafted. Furthermore, detecting these handcrafted traits necessitates technical knowledge and expertise. Moreover, these characteristics might not perfectly capture all variabilities in the sample, resulting in poorer predictive performance.

The ability of Deep Learning models to represent complicated patterns has made them a common approach for image processing. Several CNN-based methods such as ResNet, VGG-16, Inception, VGG-19, and others were proposed for image classification tasks. Ref. [76] authors employed Deep CNN for BHI classification. The authors of Ref. [8] used CNN to detect invasive BC. In contrast, the author of Ref. [77] used the same CNN approach to address the sample class imbalance and extractions of input image features at various BHIs Magnification. The authors of Ref. [78] employed the Residual neural network for automated BHI assessment. The authors of Ref. [79] combined CNN and Residual neural network for multi-level feature extraction. The authors of Ref. [80] argued for the integration of squeeze and excitation blocks and residual neural network yields compared to Ref [79] for this classification. The authors of Ref. [81] suggested that the combination of Ref. [79] and Ref. [80] yields a better result. They used Ref. [80]’s approach to extract the input image features in Latent space and used an attention mechanism [80] for classification. Transfer learning [82,83,84] has been widely investigated as it provides room for better model performance where there are few training samples. Ref [85] used Inception with a residual connection model via transfer learning for more feature extraction. Ref. [86] entails using CNN’s wavelet decomposition for image classification. Ref. [87] integrated a soft attention network to its architecture to focus entirely on the region of interest alone. At the same time, the author of Ref. [88] designed a class-specific Deep CNN network for BHIs multiclass classification. To tackle the computational cost of processing huge images, the authors of Ref. [89] developed a dual-stage CNN. The authors of Ref [90] integrated the idea of Refs. [76,86]. They used adaptive spectral composition and an attention technique [90] for classification.

Several researchers have employed the hybrid technique to seek a better and more accurate BHI classification model. The authors of Ref. [91] used the ensemble of ResNet50, VGG19 and VGG16 as feature extractors for a logistic regressor classifier. The authors of Ref. [92] suggested that a cascaded ensemble model with an SVM classifier yields better and more accurate results. The cascaded ensemble is seen at the feature extraction (multi-lateral and syntactic feature) by the CNN model. Ref. [92] created an ensemble of DenseNet121, InceptionV3, ResNet50, and VGG-16 as feature extractors. Ref. [93] investigated several Deep learning pre-trained models as feature extractors and used SVM as classifiers. Unfortunately, CNN-based techniques require a substantial amount of labeled training samples. Much research that focused on patch level [94] feature extraction and image-level [95] feature extraction for BHIs classification has been performed. The author of Ref. [95] used a voting principle for the classification after extracting input image features via image and patch levels. In contrast, the authors of Ref [94] employed pre-trained models (ResNet and Inception architecture) for input image feature extraction via images and patch level. Notwithstanding, there are chances where the input images analyzed for patch features fail to contain RIO, thus yielding false malignancy results as they might not adequately depict the input image.

Research has proposed numerous convolutional neural network-based classification architectures for BHIs to extract features from the entire input image. This approach mostly fails as the network might overlook the essential features. The identified properties/regions of the input images that might be overlooked are the cores, proliferative cells, and ducts, which are critical in determining the tumor’s malignancy. As a result, neglecting certain traits may impact outcomes. Furthermore, extracting distinctive features at different magnifications is difficult due to the tiny size of cores. To address these constraints of multiclassification of BC using the BHI dataset, this article proposes “DEEP_Pachi”, an end-to-end deep learning model incorporating multiple self-attention network heads and Multilayer Perceptron. The input images are processed as a series of patches. Each patch is squished into a single feature vector by merging the layers of all pixels in a patch and then exponentially extending it to the appropriate input dimension. Even though the proposed architectures require more training samples than CNN architectures, the most typical approach is to use a pre-trained network and then to finetune it on a smaller task sample. This paper used the option of pre-trained networks to mitigate the issues of more training sample requirements of the proposed model. To select the pre-trained networks, we first examine four pre-trained deep learning models (DensetNet201, VGG16, InceptionResNetV2, and Xception network) on BHIs images using a transfer learning technique. Afterward, an ensemble of pre-trained models functioned as feature extractors for the DEEP_Pachi network. We propose an automated method to distinguish between benign breast tumors such as Adenosis, Fibroadenoma, Phyllodes_tumor, and Tubular_adenoma and malignant breast tumors Ductal_carcinoma, Lobular_Carcinoma, Mucinous_Cancinoma, and Papillary_carcinoma to help medical diagnosis even when professional radiologists are not accessible. Furthermore, to provide a point of comparison for our findings, the proposed method is compared to other baseline models and recently published research.

The significant contribution of this paper is summarized as follows:

❖ This research reviews several Medical BC imaging techniques, their robustness and limitation, and associated public dataset.
❖ This paper proposed a fine-tuned approach termed “DEEP_Pachi,” an end-to-end deep learning model incorporating multiple self-attention network heads and Multilayer Perceptron for the multiclassification of Breast cancer diseases using histopathological images.
❖ According to the comprehensive study via transfer learning experiment, the suggested feature extractor discriminates remarkably between benign breast tumors such as Adenosis, Fibroadenoma, Phyllodes_tumor and Tubular_adenoma malignant breast tumors Ductal_carcinoma, Lobular_Carcinoma, Mucinous_Cancinoma, and Papillary_carcinoma to help medical diagnosis even when professional radiologists are not accessible.
❖ We reported a well robust deep learning method in Accuracy, Specificity, Sensitivity, Precision, F1 Score, Confusion matrix, and AUC using receiver operating characteristics (ROC) for the multiclassification of Breast cancer diseases using histopathological images based on the detailed experimental evaluation of the proposed model and comparison with state-of-the-art results.
❖ Finally, this research suggests that the proposed model “DEEP_Pachi” can also be used to increase ensemble deep learning models’ detection and classification accuracies.

The remainder of this article is organized as follows; Section 1 is devoted to the introduction and relevant studies of this research. Section 2 outlines the materials, the proposed approach, and the evaluation measures. Section 3 introduces the experimental setup and outcomes, whereas Section 4 explains the results. Section 5 discusses the conclusion and future studies.

## 2. Materials and Methods

**Figure 1:**
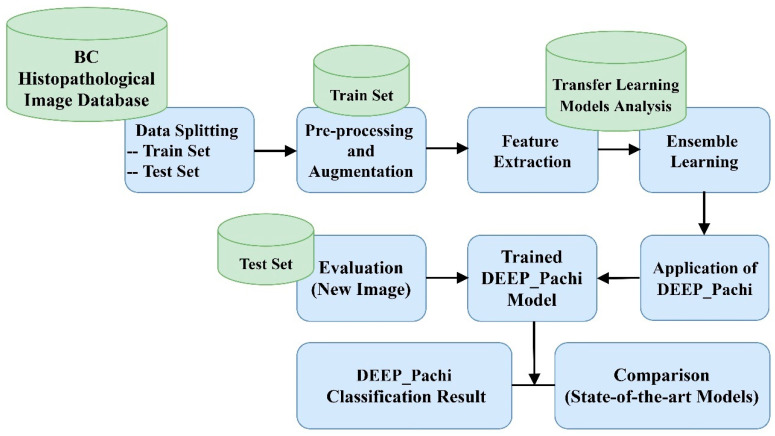
Proposed methodology block diagram.

**Figure 2:**
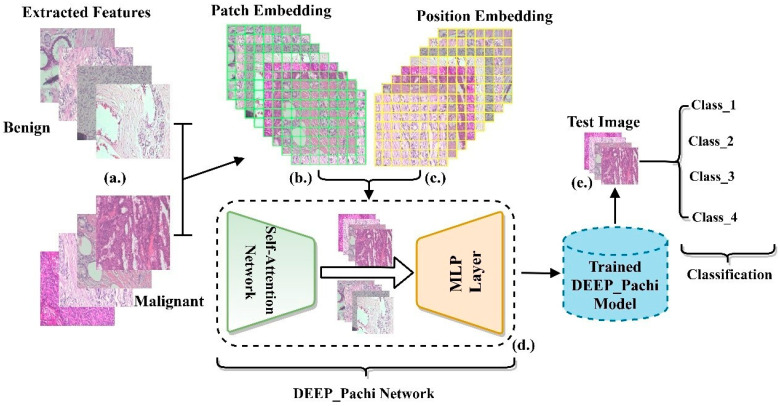
Proposed model block diagram. (**a**) depicts the extraction of input image features via the backbone models (ensemble model). The Deep_Pachi networks accepts the extracted features in two scenarios (**b**) Patch embedding and (**c**) Position Embedding. (**d**) depicts DEEP_Pachi framework components which are the self-attention Network and MLP Layer. (**e**) depicts the testing stage with new images on the trained DEEP_Pachi Network.

This section examines the suggested architecture and materials in depth. The implementation structure of this research is depicted in Figure 1. First, this paper argues that data preprocessing should only be applied to the training set because when test set data are preprocessed, there is every likelihood that the training model will perform poorly in real-time; thus, the first step in this paper was to split the dataset downloaded from the database. After splitting the dataset into train and test sets, data preparation procedures such as scaling, rotation, cropping, and normalization are performed in the train set. To make our model robust enough, transfer learning was used as the network backbone’s (feature extraction). While selecting the optimum network backbone for the proposed model, this paper conducted an experimental examination on four deep learning pre-trained models. On the other hand, researchers have argued that ensemble models provide more generalized results than single models; hence, we adopted the ensemble architecture for the proposed network backbone. The ensemble network now serves as the input to the proposed model (DEEP_Pach). The proposed model comprises a self-attention network and an MLP block, as seen in Figure 2. The self-attention network receives the input in two forms: patch embedding and position embedding. This helps the self-attention network differentiate between the various symptoms in the fed images. The multilayer perceptron (MLP) block improves the self-attention network’s outcomes in false symptom detection in the fed dataset. The input evaluated by the self-attention network is transferred to the multilayer perceptron layer for extraction before being passed to the classification/detection layer for prediction. We go over the following stages for putting our suggested approach into action.

❖ Step 1: Data collection, splitting, and data preprocessing
❖ Step 2: Backbone selection and Ensembling for more robust and generalized features. The examined models were DenseNet201, VGG16, Xception, and InceptionResNetV3 architecture.
❖ Step 3: Feeding the extracted features from the ensemble model into DEEP_Pach architecture.
❖ Step 4: This is the last stage of the proposed model: the identification and classification stage. The learned features are passed into the classification layer for the final result prediction.
❖ Step 5: Then, evaluation with the test set is performed after training.

### 2.1. Dataset

**Figure 3:**
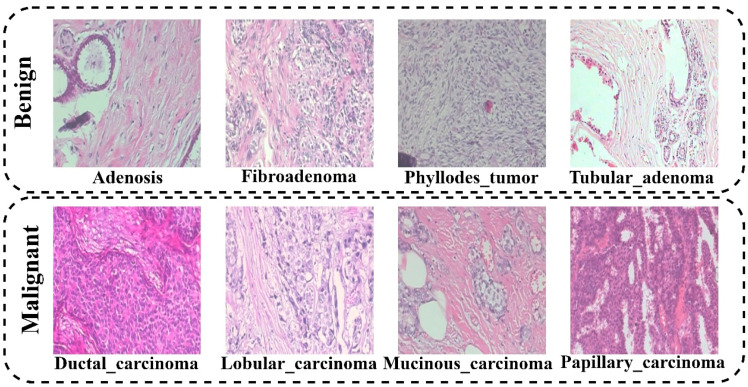
Visualization of the BreaKHis dataset.

BreaKHis, the broadest currently accessible dataset of BC histopathology images, was introduced by the authors of Ref. [4]. The dataset was obtained in brazil at the Pathological Anatomy and Cytopathology (P&D) Lab. Eighty-two patients were diagonalized, generating Benign microscopic images (BI) and Malignant images (MI) in several magnifications. The BI is 2480 in number while MI is 5429, totaling 7909 images. The generated microscopic images magnification includes 40×, 100×, 200×, and 400×. Figure 3 shows the pictorial illustration of the BreaKHis dataset. It depicts the binary classification, Benign vs. Malignant, and each class’s subclass. The benign classes include the following adenosis (A), fibroadenoma (F), phyllodes_tumor (PT), and tubular_adenoma (TA), while the malignant classes include ductal_carcinoma (DC), lobular_carcinoma (LC), mucinous_carcinoma (MC), and papillary_carcinoma (PC). Table 3 summarizes the distribution of the employed BreaKHis dataset.

### 2.2. Data Pre-Processing/Augmentation

The first step towards the employed dataset was to augment the data as the number of samples in each subclass varies. Moreover, it is worthy to note that deep learning models require a massive quantity of data to increase their performance or minimize the rate of misdetection and classification of the minority samples. Table 4 shows the type of data argumentation carried out in this paper. Augmentor is a Python library used by researchers to increase the number of samples.

The Python Augmentor library was only used on a different Python script to generate the training samples as the original samples were kept for evaluation of the model. Samples numbering 1500 were generated for training in each magnification for benign and malignant. The TensorFlow data loader function was used during training to augment the train set further. Images were rescaled (rescale operation indicates image magnification or reduction) using the 1./255 ratio: zoom range = 0.2, rotation range = 1, and horizontal flip = True. The rotation range specifies the span under which the images were spontaneously rotated throughout training. Zoom range dynamically zooms the images to a ratio of 0.2 percent, and the images were eventually flipped horizontally.

### 2.3. Network Backbone

**Figure 4:**
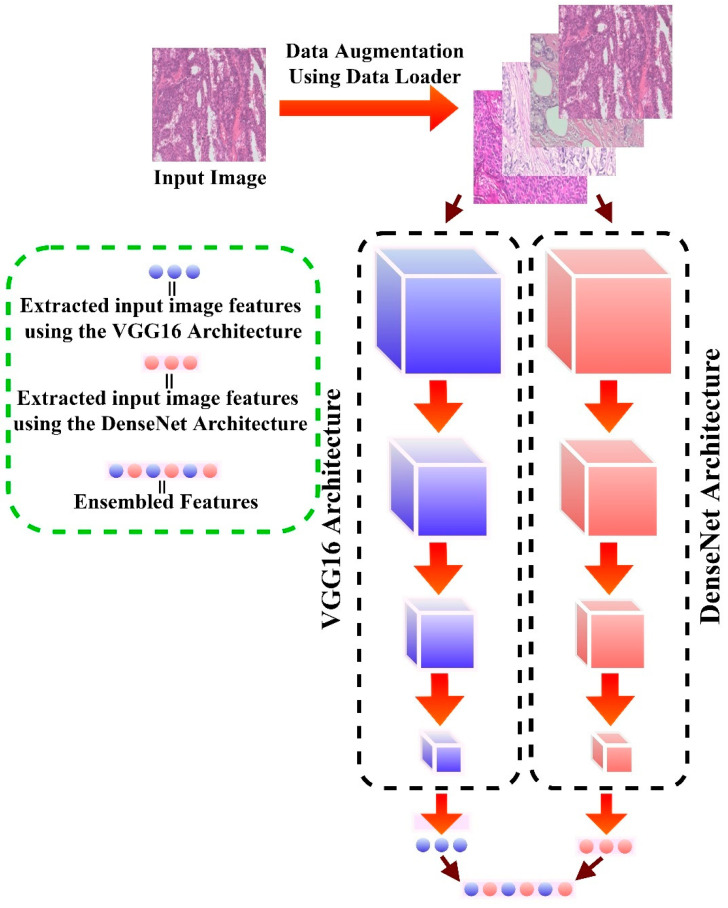
Proposed network backbone architecture.

The proposed network backbone in this study is the ensemble of two deep-learning models via the transfer learning approach. Four deep learning pretrained models were first examined using the malignant subclass magnification of the BreaKHis dataset: the DenseNet201 and the VGG16 architecture produced a better classification performance among the four examined models. Hence, we used both as the network backbone via the ensemble approach. Ensembling is the capacity to combine several learning algorithms to obtain their collective performance, i.e., to improve the performance of existing models by integrating many models into a single trustworthy model. The network backbone serves as feature extractors to the proposed model DEEP_Pachi, as seen in Figure 4.

❖ **VGG16 [96]:** VGG16 consists of 16 layers. Following preprocessing, the captured values are fed into a stacked Convolutional layer with 3 × 3 receptive-field filters and a fixed stride of 1. Following that, five max-pooling convolutional layers are used to perform spatial pooling. A 2 × 2 filter’s max-pooling layer is run with a stride of 2. To finalize the design, two fully connected layers (FC) and SoftMax (for the output) are added at the end of the final convolution.
❖ **DenseNet201 [97]:** This architecture assures information flow across network levels by linking each layer to each layer in a feed-forward fashion (with equal feature-map size). It concatenates (.) the previous layer’s output with the output of the next layer. The transition layers consist of a 1 × 1 convolution followed by a 2 × 2 average pooling. Global pooling is utilized after the last dense block before applying SoftMax.

Table 5 summarises the parameters of all implemented models in this article.

### 2.4. DEEP_Pachi Architecture

The proposed architecture is based on an attention mechanism and multilinear perceptron [98]. The attention mechanism is self-attention. The attention function is the mapping to an output of a set of keys, value pairs, and a query. The weights allocated to each value are determined by the query compatibility function with the relevant key, whereas the weighted sum of the values results in the output. Considering an input with dimension $d_{k}$ of queries and keys and dimension $d_{v}$, the dot product of all the queries with keys are computed by dividing each with $\sqrt{d_{k}}$ while using SoftMax to ascertain the weights on the values. The attention matrix contains a set of queries *Q*, keys *K*, and values *V*, which are used to compute the attention function simultaneously.

(1) $$Attention\left(Q,K,V\right)=softmax\left(\frac{QK^{T}}{\sqrt{d_{k}}}\right)V$$

**Figure 5:**
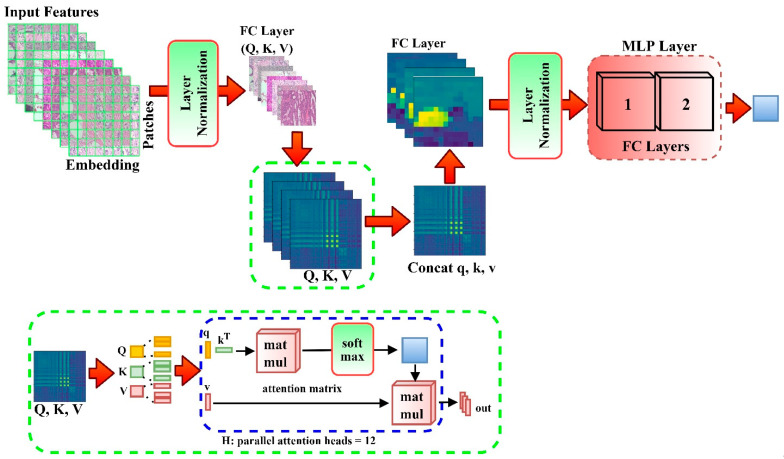
Visualization of DEEP_Pach architecture.

Multi-head attention allows the model to simultaneously attend to inputs from several representation subspaces at various locations. Figure 5 elaborates the computation performed by multi-head self-attention:

(2) $$MultiHead\left(Q,K,V\right)=Concat\left(head_{1},\cdots ,head_{h}\right)W^{O}$$

where $head_{i}=Attention\left(QW_{i}^{Q},KW_{i}^{K},VW_{i}^{V}\right)$.

The parameter matrices are projections $W_{i}^{Q}\in {\mathbb{R}}^{d_{model}*d_{k}},W_{i}^{K}\in {\mathbb{R}}^{d_{model}*d_{k}},W_{I}^{V}\in {\mathbb{R}}^{d_{model}*d_{k}}$, and $W^{O}\in {\mathbb{R}}^{hd_{i}*d_{model}}$. *MLP* is made up of two GELU non-linearity layers.

(3) $$z_{0}=\left[x_{class};x_{p}^{1}E;x_{p}^{2}E;\cdots ;x_{p}^{N}E\right]+E_{POS},\;E\in {\mathbb{R}}^{\left(p^{2\;}\times C\right)\times D},\;E_{pos}\in {\mathbb{R}}^{\left(N+1\right)\times D}\;$$

(4) $$z_{l}^{I}=MSA\left(LN\left(z_{l-1}\right)\right)+z_{l-1},\;l=1\;.\;.\;.\;.\;.L$$

(5) $$z_{l}=MLP\left(LN\left(z^{I}l\right)\right)+z_{l}^{I},\;l=1\ldots .\;L$$

(6) $$y=LN\left(z_{l}^{0}\right).$$

The classification head is implemented with one hidden layer during pre-training (Equation (5)) and a single linear layer (Equation (6)) during finetuning by an MLP. This paper uses the SoftMax layer after the MLP Block to accurately detect a sample. The SoftMax layer’s primary function converts the encoding layer’s output information into a likelihood interval (0, 1). We considered detection as a multi-classification issue in this study. After that, we send input samples to the encoding network, for which its outputs are then transferred into the likelihood interval (0, *n*) via the SoftMax layer, as seen in Equation (7):

(7) $$l_{i}=P\left(t_{i}|S_{i}\right)=\frac{1}{1+e^{-\left(W_{c}u+b_{c}\right)}}\varepsilon \left(0,n\right)$$

where the weight matrix and the bias term are denoted as $W_{c}$ and $b_{c}$, respectively. We used categorical_smooth_loss to calculate the loss between the ground truth and the detected item. Categorical_smooth_loss is the addition of smoothing of the label’s functions to the cross-entropy loss function.

### 2.5. Experimental Setup

This experiment was performed using an Intel(R) Core (TM) i9-10850K CPU @ 3.60 GHz, 64.0 GB RAM Desktop Computer, and an NVIDIA GEFORCE RTX-3080 Ti 10 GB graphics processing unit (GPU). We use open-source libraries such as Keras and TensorFlow to implement this. The experimental parameters for all of the studies documented in this work remained consistent during training: reduce learning rate (factor of 0.2, epsilon = 0.001, patience = 10, verbose = 1), es callback (early stopping, patience = 10), Adam optimizer, clip value of 0.2, and an epoch of 100. An epoch of 50 was utilized to select the pre-trained models, while all other parameters remained fixed as in the main experiment. In the encoder implementation, patch size = (2, 2), drop rate = 0.01 for all the layers, number of heads = 8, embed_dim = 64, num_mlp = 256, window size//2, and then the global average pooling for the shift size.

### 2.6. Evaluation

The proposed model used various evaluation metrics to evaluate the robustness of the model. The metrics include Accuracy, Precision, Specificity, F1-score, Sensitivity, and area under a receiver operating characteristic curve (AUC). The predefined notations are *TP* = True Positive, *FP* = False Positive, *TN* = True Negative, and *FN* = False Negative. We defined classification Accuracy (*ACC*) as follows.

(8) $$ACC=\frac{TP+TN}{\left(TP+TN\right)+\left(FP+FN\right)}\times 100$$

Precision (*PRE*) is defined as follows.

(9) $$PRE=\frac{TP}{TP+FP}\times 100$$

Specificity (SPE) is defined as follows.

(10) $$SPE=\frac{TN}{N}\times 100=\frac{TN}{TN+FP}\times 100$$

Sensitivity (*SEN*) is mathematically formulated as follows.

(11) $$SEN=\frac{TP}{P}\times 100=\frac{TP}{TP+FN}\times 100$$

The Precision and Sensitivity harmonic means are referred to as the $F_{1}\;score$, mathematically represented as thus.

(12) $$F_{1}={\left(\frac{SEN^{-1}+PRC^{-1}}{2}\right)}^{-1}=\frac{2\times TP}{2\times TP+FP+FN}$$

The AUC measures a classifier’s performance, while the probability curve is obtained from plotting at different threshold settings, the FP rate is referred to as the ROC (Receiver Operating Characteristic). The AUC indicates how well the model distinguishes between the given instances. The higher the AUC, the better. AUC = 1 implies a perfect classifier, whereas AUC = 0.5 suggests a classifier randomizing class observation. To determine the area under the ROC curve, AUC is calculated using trapezoidal integration.

## 3. Results

This section describes the results of the experiment. The parameter sensitivity experiment was first presented in this section to guide readers on how the proposed model parameter was selected for optimal performance. The transfer learning, binary, and multiclass experimental results were discussed using the employed evaluation metrics and compared with the state-of-the-art results.

### 3.1. Parameter Sensitivity Analysis of the Proposed Method

This paper carried out a parameter sensitivity analysis of the optimal number of heads and feature extractors to ascertain the parameter setting for the proposed model’s best and worst performance scenario. The number of epochs and learning rate is kept constant during this experiment. The evaluation metrics used here include accuracy, precision, and F1_score. The obtained result is recorded in Table 6. The computational cost was considered during the parameter sensitivity analysis; hence, only two, four, and eight numbers of self-attention heads and one, two, and three backbones were set up in the analysis. The backbone models used for this analysis were DenseNet201, VGG16, and Xception architecture. It was observed that using only one pre-trained network as the proposed model backbone with different numbers of self-attention heads does not have any significant result enhancement; thus, we focused on using only two and three pre-trained networks for the optimal feature selection approach. The best accuracy, F-1 score, and precision were obtained when the number of self-attention network heads is set from four using two pre-trained networks. The optimal best parameter setting of the proposed model is seen while using three pre-trained models as network backbone and setting the number of self-attention heads = 16. Although there was a minimal difference from using two pre-trained models and four self-attention heads, this paper used two pretrained model backbones and set the number of self-attention heads to be eight in all experiments to reduce the computational cost of the proposed model. The malignant class of the BreaKHis dataset was used in this evaluation. We combined all the malignant magnification subclasses into a binary classification task. We combined the 40× and the 100× magnification for low-quality image resolution while combining 200× and 400× magnification for the high-quality image resolution. We used 80 percent for training and 20% for the test during this analysis.

### 3.2. Transfer Learning Experiment for Backbone Network Selection

Having first obtaining the optimal best performance using the number of self-attention networks and number of pre-trained models for the backbone, we carried out a detailed experiment using both the Benign class and the Malignant class on various magnifications, as recorded in Table 7. From the recorded results, the transfer learning models performed very well in the benign class; hence, we focused our attention on the malignant class for backbone network selection. The excellent results of the models using the Benign class can be traced to the data preprocessing technique employed in this paper. The DenseNet201 architecture had the best result in all magnification (40×, 100×, 200×, and 400×). By comparing the recorded results, the malignant class’s results in all magnifications are lower than the benign class. VGG16 results show how robust the model is on both low and high-image resolutions compared to the Xception model. However, they recorded almost the same results in this experiment. The InceptionResNet is the least performing model; hence, DenseNet and the VGG16 were selected for the network backbone.

### 3.3. DEEP_Pachi Architecture Classification Result

**Figure 6:**
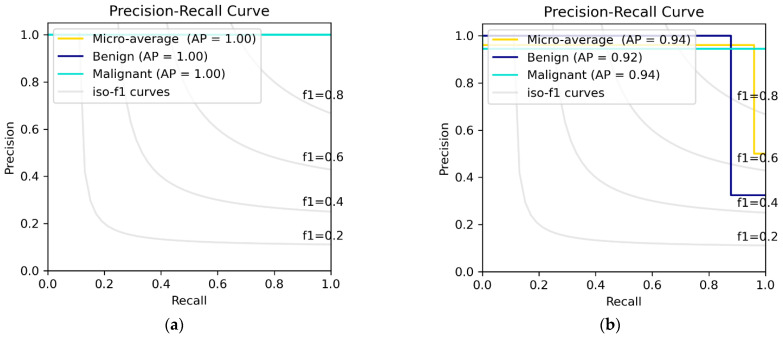

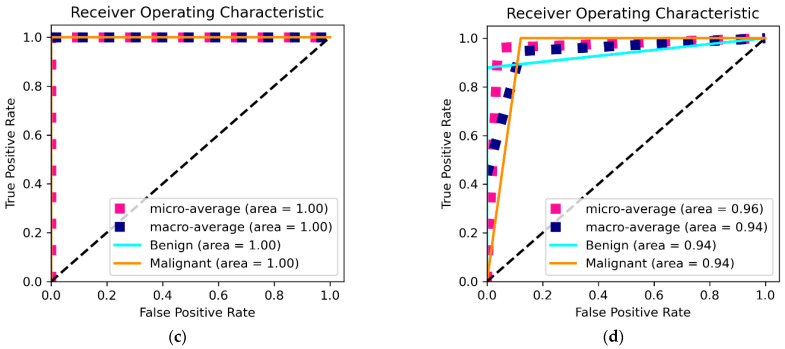
Binary Classification between Benign and Malignant. (**a**) depicts the PR Curve using the 100×, (**b**) depicts PR Curve @400× (**c**) depicts ROC curve @ 100×, and (**d**) depicts ROC curve @ 400×.

For ideal and well-detailed microscopic image analysis, the magnification factor plays a significant role; hence, this paper experimented on all BreaKHis dataset magnification (40×, 100×, 200×, and 400×). However, before then, a Binary classification was carried out on the BreaKHis dataset combing all 100× and 400× magnifications for the benign and malignant class. The reason behind selecting only the 100× and the 400× magnification was to analyze the robustness of the model in low and high-quality image resolution and have a neutral experiment without data augmentation. The binary classification is shown in Table 8. The evaluation was between the backbone network, the Ensemble of DenseNet architecture and VGG16 and the DEEP_Pachi model (Proposed model). We can see a significant contribution of the proposed model with 0.1% improvements in the Benign class and +0.1–+0.3% improvements in the Malignant class. Figure 6 visualizes the class performance of each model using the Precision–Recall curve and the Reciever Operating Characteristics (ROC) Curve.

**Figure 7:**
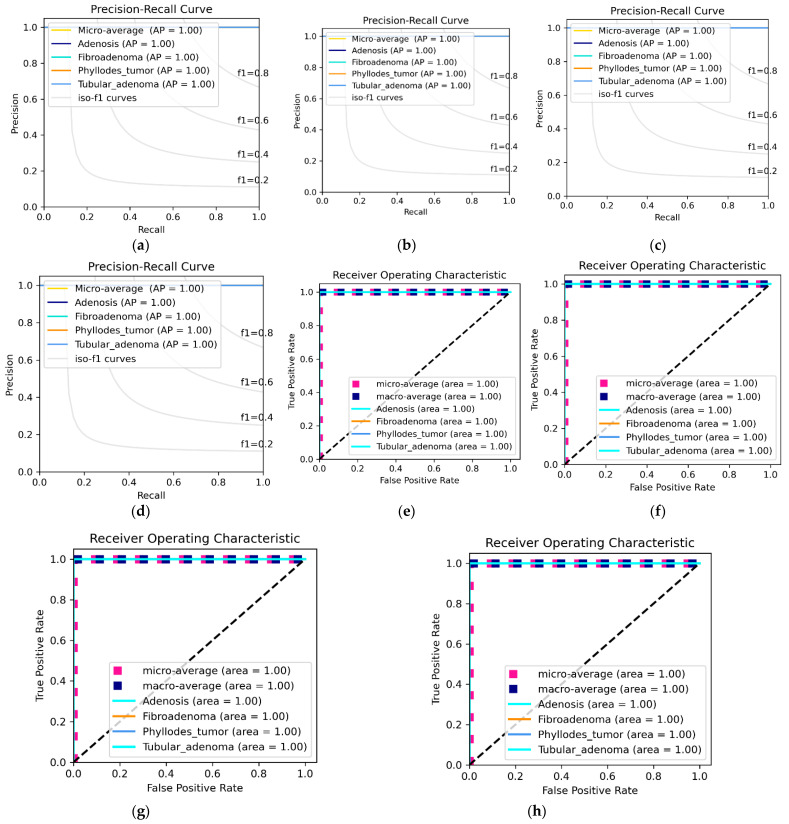
Benign individual class performance using Receiver Operating Characteristics (ROC) Curve and Precision–Recall (PR) Curve. (**a**) depicts the PR Curve @40×, (**b**) depicts PR Curve @100× (**c**) depicts PR Curve @ 200×, (**d**) depicts PR Curve @ 400×, (**e**) depicts ROC curve @ 40×, (**f**) depicts ROC curve @ 100×, (**g**) depicts ROC curve @ 200×, and (**h**) depicts ROC curve @ 400×.

**Figure 8:**
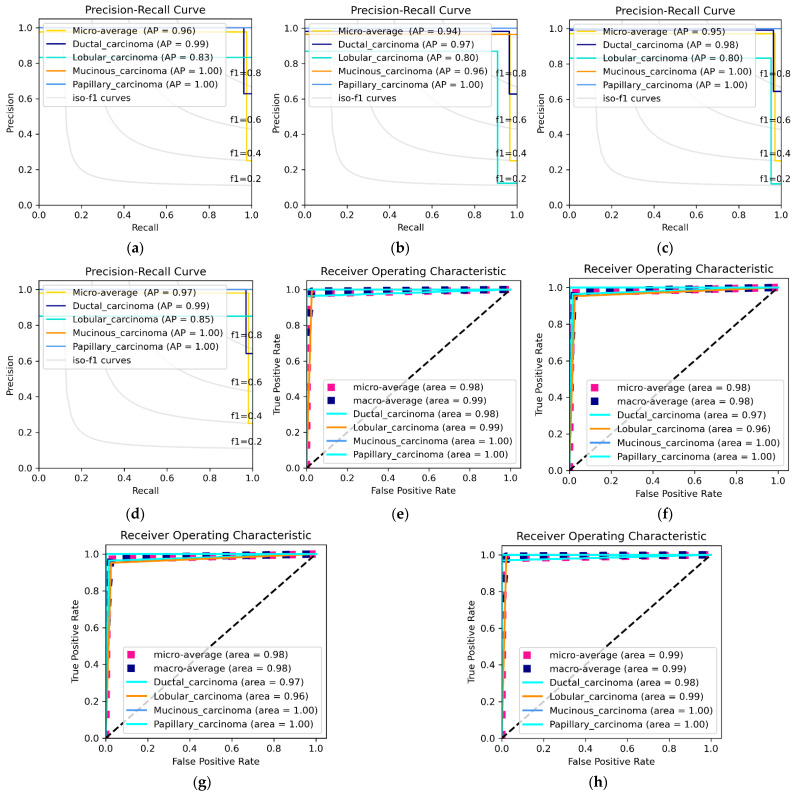
Malignant Multiclass Classification. (**a**) depicts the PR Curve @40×, (**b**) depicts PR Curve @100×, (**c**) depicts PR Curve @ 200×, (**d**) depicts PR Curve @ 400×, (**e**) depicts ROC curve @ 40×, (**f**) depicts ROC curve @ 100×, (**g**) depicts ROC curve @ 200×, and (**h**) depicts ROC curve @ 400×.

Table 9 depicts the multiclass classification of the BreaKHis dataset. Since the Benign class has described excellent results due to the ideal preprocessing techniques used in this paper, we focused our discussion more on the Malignant class. Comparing the network backbone classification performance using the Accuracy, Sensitivity, Specificity, Precision, F1-score and AUC evaluation metrics, the DEEP_Pachi architecture significantly improved by +0.1–+0.3% classification performance. Figure 7 visualized the Benign individual class performance using the Precision-Recall (PR) curve and the Reciever Operating Characteristics (ROC) Curve while Figure 8 visualized the Benign individual class performance using the Precision-Recall (PR) curve and the Reciever Operating Characteristics (ROC) Curve.

## 4. Discussion

Table 9 shows the multiclass classification performance of the proposed model vs. the backbone model (Ensemble model). Using the Precision–Recall (PR) curve and the Receiver Operating Characteristics (ROC) Curve as shown in Figure 8, the individual performances of Malignant Ductal_carcinoma, Lobular_Carcinoma, Mucinous_Cancinoma, and Papillary_carcinoma were recorded. Table 9 reveals that DEEP_Pachi classification accuracy is substantially higher than that of the Backbone model, which is four classes, with greater accuracy of at least 0.3%. These findings demonstrate that the DEEP_Pachi models significantly enhanced the accuracy of the BC classifier. These models can capture more essential tumor cell properties than traditional DL architectures. Conventional DL models comprised shallow convolution layers, which were insufficient for extracting the unique properties of BC cells, and this was a difficult task due to the significant variations of H&E staining. DEEP_Pachi models, on the other hand, can capture comprehensive information from breast types of cells, indicating the similarity of BC cells to normal breast cells. An intense network was used as our network backbone, which was critical for retaining the inherent ordering of items. In backbone models, low-level characteristics were recorded, and object pieces were retrieved at higher levels. Furthermore, the attention mechanism raises feature levels, resulting in better classification performance.

Figure 7 shows the ROC and the PR curve of the benign multiclass classification while Figure 8 shows malignant multiclass classification. The mucinous carcinoma and the papillary carcinoma attend the highest area and AP in the malignant class, whereas lobular carcinoma recorded the lowest AP and Area. Table 9 shows that when the results of the DEEP_Pachi architecture are compared to the state-of-the-art results, the backbone model alone achieves a higher accuracy for the multiclassification task. The accuracy of the backbone model alone was at least 3% greater than any of the state-of-the-art models. This demonstrates that this model can use the deep network architecture of multi-resolution input images to collect multi-scale relevant information and the benefits of its single models. The DEEP_Pachi model outperforms the multiclass classification by a margin for binary classification. This is because the various classes are not dissimilar and share many characteristics. The findings show that the backbone model outperformed the other algorithms in the binary classification task, with a total accuracy of 99%. Table 9 also shows the backbone model’s sensitivity, Sensitivity, Precision, F1-Score, and AUC vs. the DEEP_Pachi. Because our model can capture multi-level and multi-scale data and distinguish individual nucleus features and hierarchical organization, the DEEP_Pachi performed well. DEEP_Pachi may also learn features at multiple sizes through its convolutional layers. As a result, it can accurately distinguish individual nuclei and nuclei structures. The experimental findings reveal that the ensemble technique outperforms all other approaches, achieving gains of at least 0.2–0.8% for images at 40×, 100×, 200×, and 400× magnification due to its capacity to collect multi-scale contextual information. DEEP_Pachi demonstrates that features derived from cross image inputs and then merged into a boosting framework outperform standard deep learning architectures in object classification tests. This also indicates that our enhancing approach exceeds deep learning networks when dealing with few training data samples.

### 4.1. Visualization the Influence of DEEP_Pachi Framework

**Figure 9:**
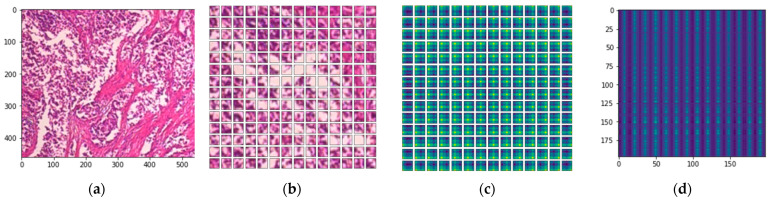
The visualization of the implementation steps of the DEEP_Pachi model. (**a**) depicts the input image, (**b**) the input image patches, (**c**) learnable position embedding of the input image patches, and (**d**) attention matrix.

To evaluate the influence of patches and embedding in the DEEP_Pachi model, an experiment was carried out utilizing the malignant image with 200× magnification as shown in Figure 9. The input image (a) was first split into patches as shown in (b) before the positional embedding (c) is added. By combining the pixel layers in a patch and then immensely extending it to the suitable input dimension, each patch is squeezed into a vector representation. Positional embedding (c) demonstrates how the model understands when to encrypt distance within the input image in the comparability of position embeddings, i.e., relatively close patches have much more position similar embeddings. The reason for the patches and the learnable embeddings is to treat each patch separately for an accurate feature extraction. The positional embedding helps the model to know where each patch was at the initial input during the output. The patches are first converted using 2D learnable convolutions. Furthermore, to analyze the impact of the patch and embedding combination, (d) validates the envisaged approach’s efficacy in improving prospective ROIs; this enalbes the model in efficiently and successfully concentrating on these areas and for determining the cancer.

**Figure 10:**
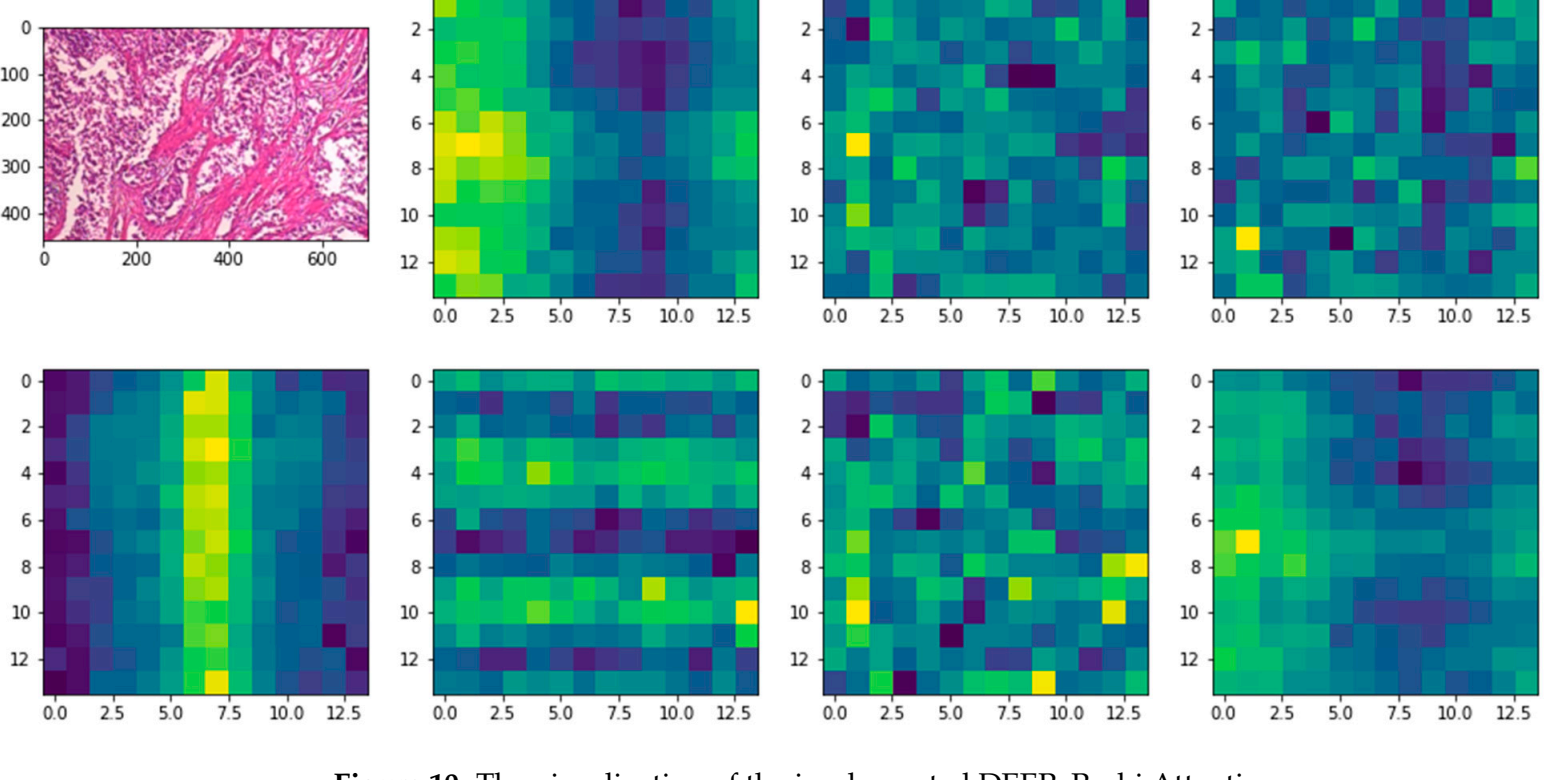
The visualization of the implemented DEEP_Pachi Attention.

Figure 9d shows how the self-attention heads enable DEEP_Pachi to generalize across the input frame, even within the minimum layers. According to the diagram, the total distance in input images in which relevant data are assimilated is comparable to receptive scale factor in CNNs and is highly recognized in our model due to our network backbone, which is an ensemble of DenseNet201 and VGG16; thus, we observed continuously small attention scales in small layers. Implementing the DEEP_Pachi model without a network backbone, i.e., generating features from scratch, causes the attention heads to focus on the majority of the image in the lowest layers, demonstrating that the model’s potential to consolidate information globally really is used. Furthermore, as the network depth increases, so does attention proximity. We discover that the model focuses on visual features that are semantic information significant for classification, as depicted in Figure 10.

### 4.2. Comparison with the State-of-the-Art Results

This section discusses the proposed model results vs. the state-of-the-art results. The result is illustrated in Table 10. The state-of-the-art models can be seen in two approaches—single models and ensemble models. Ensemble modeling is the most general approach, as seen in Table 10. Refs. [98,99] experimented with several deep learning models as feature extractors while using conventional machine learning algorithms (SVM and LR) as classifiers. However, the results were not as promising as the recorded results are below 90%. Among well-known Deep learning models, DenseNet and Xception architectures are preferred over the other models. They tend to yield classification accuracies above 90%, as recorded in Refs. [77,100,101] suggested that extracting breast cancer features using different feature extractors boosts models’ classification performance. They employed the Shearlet-based features extractor and histogram-based features extractor. For their final models, they concatenated the output features and achieved better performance compared to single feature extractors. They performed a +5–8% accuracy improvement in all magnifications of the BreaKHis dataset Ref [102], although the result is not promising, and using Data augmentation for better performance is suggested. They carried out a binary classification of the BreaKHis dataset and a multiclass classification using 400x magnification. Among their employed data augmentation techniques, GAN-based DA yielded 77.3% accuracy for binary classification while yielding 78.5% multiclass classification performance. Comparing the performance of the inception models, Inception_V3 and Inception_ResNet_V2 [93] produced a better performance as they extracted more relevant information by running convolution operations with varied regions of interest concurrently. The use of transfer learning is more evident in binary classification. The authors of Refs. [103,104,105,106,107] based their work on binary classification by combining the subclasses of the benign and the malignant. VGG is seen to be often used for feature extraction as it has deeper layers able to identify conceptual features. Comparing our proposed model DEEP_Pachi, which is a modification of the vison transformer self-attention heads computation techniques, ensemble models, and a classification layer using the Multilinear perceptron block, we argue that extracting increased breast cancer features requires an accurate vision system and, hence, and attention mechanism to focus on the region of the disease instead of extracting entire image features. Refs. [108,109,110,111] proposed an accurate and more unique approach for breast cancer classification. Ref. [108] employed the use of multi-view attention mechanism. Ref. [109] proposed the deep attention high order network, while Ref [110] proposed using a different branch of CNN for more feature generation. Ref [111] proposed a three-channel feature low dimension model. All these approaches were in line with better breast cancer feature extraction; thus, they achieved the highest classification performance with +95% classification accuracy on all magnifications of BreaKHis (40×, 100×, 200×, and 400× magnification). In line with the current state-of-the-art results, our model achieved an accuracy of 99% for all magnifications except 400%, where we achieved an accuracy of 1.0%. Our analyses demonstrate that our proposed models significantly enhanced the efficiency of the BC classifier. Our models can extract more critical breast cell features than CNN. CNN was made up of four thin convolution layers, which were insufficient for extracting unique properties of BC tumors, which was a difficult task due to the large variation of H&E smears.

The proposed model was also evaluated using the ICIAR 2018 breast cancer Histology images used for the BACH Grand challenge [123]. This dataset has 400 images while having 100 images per class. The classes of the dataset are Normal, Benign, In situ carcinoma, and Invasive carcinoma. This paper first augmented the dataset following the same principle of augmentation used for the BreaKHis data implemented. Table 11 summarizes the result attend with that of the state-of-art results. The use of the ensemble model is very evident in the compared models. Our proposed model supersedes the accuracy of the compared models, showing our model’s superiority.

## 5. Conclusions

To tackle the extraction of irrelevant features by conventional deep learning models, which results in the model’s misclassification and prediction, this paper proposed the DEEP_Pachi framework based on ensemble model, multiple self-attention heads, and multilinear perceptron for an accurate breast cancer histological image classification. First, a thorough review of medical image modalities for breast cancer classification was carried out with the related open access datasets. Secondly, we applied the Python augmentation library to address the issues of limited raining data samples. The Python Augmentor was used to generate the training image samples while utilizing the original image for testing. The proposed model utilizes ensemble model (Densenet201 and VGG16) as the network backbone for a more generalized feature extraction of the input images (global features), whereas multiple self-attention heads extract spatial information (regions of interest). The superiority of the proposed model was evaluated using two publicly available databases, BreakHis and ICIAR2018, and using various evaluations metrics, and the result obtained show that the proposed DEEP_Pachi outperforms the state-of-the-art results in histopathological breast cancer image classification. The suggested technique achieved an accuracy of 1.0 for the benign class and 0.99 for the malignant class in all magnifications of the BreakHis datasets and an accuracy of 0.99 on the ICIAR 2018 Challenge dataset.

As much as the proposed framework exhibit high classification accuracy, there is still room to evaluate DEEP_Pachi using other data augmentation techniques. Future work will see the exploration of various data augmentation techniques such as GAN for increasing training samples. We also intend on extending the DEEP_Pachi framework to other disease classification using histopathological or microscopic images such as Oral cancer, Skin Cancer, etc. On the other hand, this paper will investigate the replacement of the MLP Block with SGTM neural-like structures to evaluate the possible best approach in our model.

## Figures and Tables

**Table 1:** Robustness and constraints of various imaging techniques for BC diagnosis and treatment.

Imaging Techniques	Robustness	Constraints	Public Datasets
MG	1. Reliable and premium approach for capturing, storing, and processing images of breast tissue [52,53] 2. Unlike HP images, they do not need a comprehensive experience or professional understanding to analyze and classify.	1. Due to their microscopic dimensions and scattered form features, they have restricted abilities in acquiring segments and sub in the human breast [54]. 2. Unsuitable for detecting breast cancer in thick breasts due to the absence of malignant tissues [55]. 3. Not reliable in identifying BC; hence more screening may be necessary for accurate assessments [56].	-BCDR -CBIS-DDSM -MIAS -Mini-MIAS -DDSM -InBreast
US	1. Does not make patients vulnerable to dangerous rays and is thus regarded exceedingly safe, particularly for expectant mothers [57]. 2. These are specifically convenient imaging techniques for identifying BC in thick breasts, where MGs fail [58]. 3. Allows for viewing a breast tumor from multiple viewpoints and configurations, lowering the possibility of a negative result assessment.	1. Often yield false diagnoses if the scanner probe is not moved or pushed appropriately [59]. 2. They cannot correctly portray the tumor outline in the breast due to its signal weakness to the human muscles [60]. 3. US images are of low quality compared to the images of MGs; thus, obtaining ROI for more advanced analysis is challenging with US imaging.	-BCDR -BUSI
MRI	1. MRI can detect questionable spots, which can be explored further with autopsy (MRI-assisted biopsy). 2. MRI, just like US, does not make patients vulnerable to any dangerous radioactive materials. 3. MRI gives a thorough description of soft breast internal tissues as well as the ability to record	1. To improve MRI images, supplement chemicals are frequently administered, which might cause sensitivities or other issues and are thus not suggested for patients, particularly renal patients [61]. 2. MRI is typically not suggested throughout pregnancy [62] and is primarily advised as a follow-up test only after an MGs-based examination has been performed. 3. MRI is a pricey procedure relative to MGs or US; hence, it is not often used for BC diagnosis. MRI offers highly accurate data about the interior breast tissues, but it can overlook some malignant areas that MGs can identify [63].	Duke-Breast-Cancer RIDER Breast MRI
HP	1. Images of HP are RGB images that are very efficient in diagnosing many types of malignancies and provide a greater efficacy for an early phase of BC. 2. An in-depth study of breast tissues is feasible with HP images, resulting in a more reliable examination of BC than other imaging alternatives. 3. Multi ROI images may be produced from full flip HP images, increasing the likelihood of detecting cancer tissues and lowering the number of false positives.	1. HP images are obtained by mammogram, which is an expensive approach with significant potential complications, necessitating special attention from pathologists as comparable to other imaging alternatives 2. HP images are easy to misinterpret, and the conventional examination of HP images takes a long time [64]. As a result, experts are needed for correct interpretation. 3. Extreme caution is required during histopathology specimen preparation (From the extraction of a tissue sample from the breast to the application of microscope to the extracted tissue sample, the adjustment/control of the color disparities caused by different staining processes) to reduce the possibility of a mistaken diagnosis.	UCI (Wisconsin) BICBH BreakHis
Identified Public site for BC Dataset	http://peipa.essex.ac.uk/info/mias.html, http://marathon.csee.usf.edu/Mammography/Database.html, https://biokeanos.com/source/INBreast, https://bcdr.ceta-ciemat.es/information/about https://wiki.cancerimagingarchive.net/display/Public/, https://www.repository.cam.ac.uk/handle/1810/250394, accessed on 20 March 2022.		

**Table 2:** Summary of the related studies.

Ref	Year	Image Type	Techniques	Task	Recorded Result
[8]	2017	-	ConvNet classifier	Detection	75.86% Dice coefficient 71.62% positive prediction 96.77% negative prediction (pixel-by-pixel evaluation)
[12]	2017	-	Multiscale Basic Image Features, Local Binary Patterns, Random Decision Trees Classifier	Classification	84% Accuracy
[32]	2017	BreaKHis Augmented BreaKHis	CSDCNN model	Multi-Classification	93.2% accuracy
[37]	2017	-	Hybrid Contour Model-Based Segmentation with SVM Classifier	Binary Classification Multi-Classification	88% AUC.
[36]	2018	BreaKHis	VGG16, VGG19, and ResNet50 with Logistic Regression	Binary Classification	92.60% accuracy, 95.65% AUC, 95.95% precision score
[33]	2018	BACH (ICIAR 2018)	Two-Stage CNN	Multi-Classification	95% accuracy
[4]	2018	BreaKHis	DL model with handcrafted features	Mitosis detection	92% Precision 88% Recall 90% F-Score
[5]	2018	BreaKHis	Transfer Learning based CNN	Mitosis detection	15% F1-Score improvement
[27]	2018	TMAD, OUHSC	Transfer Learning.	Binary Classification	90.2% Accuracy with GoogleNet
[23]	2019	BACH (ICIAR 2018)	Hybrid CNN + Deep RNN	Multi-Classification	91.3% Accuracy
[24]	2019	BreaKHis	Small SE-ResNet	Binary Classification Multi-Classification	98.87–99.34% Binary Classification Accuracy 90.66–93.81% Multi-Classification Accuracy
[25]	2019	BACH (ICIAR 2018) Bioimaging2015 Extended Bioimaging2015	CNN + RNN + Attention Mechanism	Multi-Classification	-
[6]	2019	BreaKHis	Mask R-CNN network, with features obtained from Handcrafted and DCNN	Mitosis detection	-
[26]	2019	BreaKHis L.R.H. hospital Peshawar Data	Transfer Learning. GoogleNet, VGGNet, ResNet	Binary Classification	97.53% Accuracy
[28]	2019	BreaKHis	D^2^TL and ICELM	Binary Classification	Classification Accuracy 96.67%, 96.96%, 98.18%
[29]	2019	BreaKHis	Inception_V3 Inception_ResNet_V2	Multi-Classification	-
[30]	2019	BreaKHis BACH (ICIAR 2018)	Deep CNN with Wavelet decomposed mages	Binary Classification Multi-Classification	96.85% Accuracy 98.2% Accuracy
[34]	2019		deep selective attention	Classification	98% accuracy
[21]	2020	B.H.I.s BreaKHis	Modified Inception Network/Transfer Learning	Classification multiclass	-
[22]	2020	BreaKHis	ResHist model (Residual Learning CNN)	Classification	84.34% Accuracy 90.49% F1-Score 92.52% Accuracy (DA) 93.45% F1-score (DA)
[31]	2020	BACH (ICIAR 2018)	Attention Guided CNN	Detection and Classification	90.25 ± Accuracy 0.98425 AUC Single 88% Accuracy Ensemble 93% Accuracy
[35]	2020	BreaKHis BACH (ICIAR 2018)	CNN and multi-resolution Spatial Features wavelet transform	Binary Classification Multi-Classification	97.58% Accuracy 97.45% Accuracy
[38]	2020	BreaKHis	CNN With Several Classifiers	Binary Classification	
[39]	2020		VGG16, VGG19, and ResNet50 with SVM		
[19]	2021	BHIs	DCNN with several Optimizers	Classification	99.05% accuracy

**Table 3:** BreaKHis dataset.

Class	Sub_Class	Magnification				Total	Nos_Patients
		40×	100×	200×	400×		
Benign	Adenosis	114	113	111	106	444	24
	Fibroadenoma	253	260	264	237	1014	
	Phyllodes_tumor	109	121	108	115	453	
	Tubular_adenoma	149	150	140	130	569	
Malignant	Ductal_carcinoma	864	903	896	788	3451	58
	Lobular_carcinoma	156	170	163	137	626	
	Mucinous_carcinoma	205	222	196	169	792	
	Papillary_carcinoma	145	142	135	138	560	
Total		1995	2081	2013	1820	7090	82

**Table 4:** Data augmentation Python algorithm.

Import Augmentor
def upsample(dir, num_samples):
p = Augmentor.Pipeline(dir)
p.rotate(probability = 1, max_left_rotation = 5, max_right_rotation = 5)
p.zoom(probability = 0.2, min_factor = 1.1, max_factor = 1.2)
p.skew(probability = 0.2)
p.shear(probability = 0.2, max_shear_left = 2, max_shear_right = 2)
p.crop_random(probability = 0.5, percentage_area = 0.8)
p.flip_random(probability = 0.2)
p.sample(num_samples)
p.random_distortion(probability = 1, grid_width = 4, grid_height = 4, magnitude = 8)
p.flip_left_right(probability = 0.8)
p.flip_top_bottom(probability = 0.3)
p.rotate90(probability = 0.5)
p.rotate270(probability = 0.5)
src_dir = ‘D:/Pachigo/Breast_Cancer/Train/Benign/40
src_dir = ‘D:/Pachigo/Breast_Cancer/Train/Benign/100
src_dir = ‘D:/Pachigo/Breast_Cancer/Train/Benign/200
src_dir = ‘D:/Pachigo/Breast_Cancer/Train/Benign/400
upsample(src_dir, 1500)

**Table 5:** Optimal parameters of all implemented models.

Models	Learning Rate	Loss Function	Trainable Parameter	Non-Trainable Parameter	Total Parameter	Optimizers	Nos. of Epochs
DenseNet201	0.001	Categorical smooth loss	1,106,179	18,321,984	19,428,163	Adam	Early stop
VGG16	0.001	Categorical smooth loss	598,403	14,714,688	15,313,091	Adam	Early stop
InceptResNetV2	0.001	Categorical smooth loss	393,475	54,336,736	54,730,211	Adam	Early stop
Xception	0.001	Categorical smooth loss	1,179,907	20,861,480	22,041,387	Adam	Early stop
Ensemble	0.001	Categorical smooth loss	43,872,899	33,036,672	76,909,571	Adam	Early stop
DEEP_Pachi	0.001	Categorical smooth loss	766,291	33,036,848	33,803,139	Adam	Early stop

**Table 6:** Parameter sensitivity analysis of DEEP_Pachi.

Nos. of Pre-Trained Network	Nos. of Self-Attention Heads	Learning Rate	Nos. of Epoch	Accuracy (%)	Precision (%)	F1_Score (%)
1	2	3 × 10^−3^	50	0.96	0.96	0.96
2	2	3 × 10^−3^	50	0.96	0.97	0.96
3	2	3 × 10^−3^	50	0.97	0.97	0.97
1	4	3 × 10^−3^	50	0.96	0.97	0.96
2	4	3 × 10^−3^	50	0.97	0.98	0.97
3	4	3 × 10^−3^	50	0.98	0.97	0.97
1	8	3 × 10^−3^	50	0.96	0.97	0.97
2	8	3 × 10^−3^	50	0.97	0.99	0.98
3	8	3 × 10^−3^	50	0.98	0.98	0.98
1	16	3 × 10^−3^	50	0.98	0.98	0.98
2	16	3 × 10^−3^	50	0.99	1.0	0.98
3	16	3 × 10^−3^	50	1.0	0.98	0.99

**Table 7:** Transfer learning classification result. The experiment was performed specifically for the selection of the proposed model backbone.

Models	ACC (%)	SEN (%)	SPE (%)	PRE (%)	F1_Score (%)	AUC (%)
40× Magnification-Benign						
DenseNet201	1.0	1.0	1.0	1.0	1.0	1.0
InceptionResNet	0.99	0.99	0.99	0.98	0.98	0.99
VGG16	1.0	1.0	1.0	1.0	1.0	1.0
Xception	1.0	1.0	1.0	1.0	1.0	1.0
100× Magnification-Benign						
DenseNet201	1.0	1.0	1.0	1.0	1.0	1.0
InceptionResNet	1.0	1.0	1.0	1.0	1.0	1.0
VGG16	0.99	0.99	0.99	0.98	0.98	0.99
Xception	0.99	0.99	0.99	0.98	0.98	0.99
200× Magnification-Benign						
DenseNet201	1.0	1.0	1.0	1.0	1.0	1.0
InceptionResNet	0.99	0.98	0.99	0.99	0.98	0.98
VGG16	1.0	1.0	1.0	1.0	1.0	1.0
Xception	1.0	1.0	1.0	1.0	1.0	1.0
400× Magnification Benign						
DenseNet201	1.0	1.0	1.0	1.0	1.0	1.0
InceptionResNet	1.0	1.0	1.0	1.0	1.0	1.0
VGG16	0.99	0.98	0.99	0.99	0.98	0.98
Xception	0.99	0.98	0.99	0.99	0.98	0.98
40× Magnification Malignant						
DenseNet201	0.98	0.99	0.99	0.95	0.97	0.99
InceptionResNet	0.94	0.95	0.97	0.83	0.88	0.96
VGG16	0.94	0.93	0.96	0.82	0.86	0.94
Xception	0.94	0.93	0.96	0.82	0.86	0.94
100× Magnification Malignant						
DenseNet201	0.97	0.98	0.98	0.91	0.94	0.98
InceptionResNet	0.94	0.95	0.97	0.83	0.88	0.96
VGG16	0.94	0.94	0.96	0.83	0.87	0.95
Xception	0.96	0.96	0.97	0.86	0.90	0.97
200× Magnification Malignant						
DenseNet201	0.98	0.97	0.98	0.94	0.95	0.98
InceptionResNet	0.93	0.94	0.96	0.80	0.85	0.95
VGG16	0.92	0.93	0.95	0.79	0.84	0.94
Xception	0.95	0.95	0.97	0.85	0.89	0.96
400× Magnification Malignant						
DenseNet201	0.98	0.98	0.98	0.92	0.95	0.98
InceptionResNet	0.96	0.97	0.98	0.88	0.92	0.97
VGG16	0.97	0.96	0.98	0.90	0.93	0.97
Xception	-	-	-	-	-	-

ACC denotes Accuracy; SEN = Sensitivity; SPE = Specificity; PRE = Precision; AUC = Area under the ROC Curve.

**Table 8:** Binary classification using DEEP_Pachi.

Models	ACC (%)	SEN (%)	SPE (%)	PRE (%)	F1_Score	AUC
100× Magnification						
Backbone Network	0.99	0.99	0.99	0.99	0.99	0.99
DEEP_Pachi	1.0	1.0	1.0	1.0	1.0	1.0
400× Magnification						
Network Backbone	0.95	0.93	0.93	0.95	0.94	0.93
DEEP_Pachi	0.96	0.96	0.96	0.97	0.95	0.96

**Table 9:** Multiclass classification using DEEP_Pachi vs. the network backbone.

Models	ACC (%)	SEN (%)	SPE (%)	PRE (%)	F1_Score (%)	AUC (%)
40× Magnification-Benign						
Network Backbone	1.0	1.0	1.0	1.0	1.0	1.0
DEEP_Pachi	1.0	1.0	1.0	1.0	1.0	1.0
100× Magnification-Benign						
Network Backbone	1.0	1.0	1.0	1.0	1.0	1.0
DEEP_Pachi	1.0	1.0	1.0	1.0	1.0	1.0
200× Magnification-Benign						
Network Backbone	1.0	1.0	1.0	1.0	1.0	1.0
DEEP_Pachi	1.0	1.0	1.0	1.0	1.0	1.0
400× Magnification Benign						
Network Backbone	1.0	1.0	1.0	1.0	1.0	1.0
DEEP_Pachi	1.0	1.0	1.0	1.0	1.0	1.0
40× Magnification Malignant						
Network Backbone	0.97	0.98	0.98	0.92	0.94	0.98
DEEP_Pachi	0.99	1.0	1.0	0.96	0.98	0.98
100× Magnification Malignant						
Network Backbone	0.97	0.98	0.98	0.91	0.94	0.98
DEEP_Pachi	0.99	1.0	1.0	0.94	0.98	0.98
200× Magnification Malignant						
Network Backbone	0.96	0.96	0.98	0.90	0.92	0.97
DEEP_Pachi	0.99	0.99	0.99	0.95	0.98	0.98
400× Magnification Malignant						
Network Backbone	0.98	0.98	0.98	0.92	0.95	0.98
DEEP_Pachi	1.0	1.0	1.0	0.97	0.99	0.99

**Table 10:** Result comparison with the state-of-the-art result using the BreaKHis Dataset.

Ref/Year	Approach	Data Type	Classification Type	Accuracy (%)				
				40×	100×	200×	400×	Binary
[112] 2018	Ensemble (CNN + LSTM)	BreaKHis		88.7	85.3	88.6	88.4	
[113] 2018	DenseNet CNN	BreaKHis		93.6	97.4	95.9	94.7	
[77] 2018	Xception	BreaKHis		95.3	93.4	93.1	91.7	
[114] 2018	KAZE features + Bag of Features	BreaKHis		85.9	80.4	78.1	71.1	
[102] 2019	CNN	BreaKHis						77.2
	CNN + DA							76.7
	CGANs based DA							77.3
	DA + CGANs based DA							75.2
	CNN						75.4	
	CNN + DA						75.9	
	CGANs based DA						78.5	
	DA + CGANs based DA						78.7	
[115] 2019	Deep ResNet + CBAM	BreaKHis		91.2	91.7	92.6	88.9	
[103] 2019	Transfer Learning (VGG16 + VGG19 + CNN)			98.2	98.3	98.2	97.5	
								98.1
[116] 2019	IRRCNN	BreaKHis		98.0	97.6	97.3	97.4	
[85] 2019	Inception_V3	BreaKHis	Multiclass	90.3	85.4	84.0	82.1	
			Binary	97.7	94.2	87.2	96.7	
	Inception_ResNet_V2		Multiclass	98.4	98.7	97.9	97.4	
			Binary	99.9	99.9	1.0	99.9	
[80] 2019	BHCNet-6 + ERF	BreaKHis	Multiclass	94.4	94.5	92.3	91.1	
	CNN +SE-ResNet		Binary	98.9	99.0	99.3	99.0	
[117] 2020	Deep CNN	BreaKHis		73.4	76.8	83.2	75.8	
[94] 2020	VGG16 + SVM (Balanced + DA)	BreaKHis		94.0	92.9	91.2	91.8	
	Ensemble (VGG16 + VGG19 + ResNet 50) + RF Classifier			90.3	90.1	87.4	86.6	
	Ensemble (VGG16 + VGG19 + ResNet 50) + SVM Classifier			82.2	87.6	86.5	83.0	
[78] 2020	ResHist (RL Based 152-layer CNN)	BreaKHis		86.4	87.3	91.4	86.3	
[64] 2020	VGGNET16-RF	BreaKHis		92.2	93.4	95.2	92.8	
	VGGNET16-SVM			94.1	95.1	97.0	93.4	
[118] 2020	CNN + spectral–spatial features	BreaKHis	Malignant	97.6	97.4	97.3	97.0	
[100] 2020	NucTraL+BCF	BreaKHis						96.9
[119] 2020	ResNet50 + KWE LM	BreaKHis	Malignant	88.4	87.1	90.0	84.1	
[93] 2020	AlexNet + SVM	BreaKHis		84.1	87.5	89.4	85.2	
	VGG16 + SVM			86.4	87.8	86.8	84.4	
	VGG19+SVM			86.6	88.1	85.8	81.7	
	GoogleNet + SVM			81.0	84.5	82.5	79.8	
	ResNet18 + SVM			84.0	84.3	82.5	79.8	
	ResNet50 + SVM			87.7	87.8	90.1	83.7	
	ResNet101 + SVM			86.4	88.9	90.1	83.2	
	ResNetInceptionV2 + SVM			86.3	86.3	87.1	81.4	
	InceptionV3 + SVM			85.8	84.7	86.8	82.9	
	SqueezeNet + SVM			81.2	83.7	84.2	77.5	
[120] 2020	Optimized CNN	BreaKHis		80.8	76.6	79.9	74.2	
[110] 2020	InceptionV3 + BCNNs	BreaKHis		95.7	94.7	94.8	94.5	
								96.1
[105] 2020	VGG16 + SVM	BreaKHis		78.6	85.2	82.0	79.6	
	VGG19 + SVM			77.3	79.1	83.0	79.1	
	Xception + SVM			81.6	82.9	78.4	76.1	
	ResNet50 + SVM			86.4	86.0	84.3	82.9	
	VGG16 + LR			78.8	85.2	81.2	79.1	
	VGG19 + LR			77.6	82.4	82.2	77.8	
	Xception + LR			82.4	79.6	79.4	83.1	
	ResNet50 + LR			83.1	86.7	84.0	80.1	
[107] 2020	Shearlet-based features	BreaKHis		89.4	88.0	86.0	83.0	
	Histogram-based features.			92.6	93.9	95.0	94.7	
	Concatenating all features			98.2	97.2	97.8	97.3	
[104] 2021	MA-MIDN	BreaKHis		96.3	95.7	97.0	95.4	
[108] 2021	AhoNet (Resnet18 + ECA + MPN-COV)	BreaKHis		97.5	97.3	99.2	97.1	
[109] 2021	3PCNNB-Net	BreaKHis		92.3	93.1	97.0	92.1	
[121] 2021	APVEC	BreaKHis		92.1	90.2	95.0	92.8	
[111] 2021	Stochastic Dilated Residual Ghost Model	BreaKHis		98.4	98.4	96.3	97.4	
[105] 2021	Transfer Learning via Fine-tuning Strategy	BreaKHis		99.3	99.0	98.1	98.8	
								98.4
[122] 2021	BCHisto-Net	BreaKHis	100× Magnification					89
Ours	DEEP_Pachi	BreaKHis		99.8	99.8	99.8	1.0	99.8

**Table 11:** Result comparison with the state-of-the-art result using the ICIAR 2018 Dataset.

Ref/Year	Approach	Data Type	Accuracy (%)
[18] 2018	DCNN + SVM	BACH	77.8
[123] 2018	Pre-trained VGG-16	BACH	83.0
	Ensemble of three DCNNs		87.0
[124] 2018	Ensemble (DenseNet 169 + Denseness 201 + ResNet 34)	BACH	90.0
[20] 2019	All Patches in One Decision	BACH	90% 92.5
[125] 2019	Ensemble (DenseNet 161+ ResNet 152 + ResNet 101)	BACH	91.8
[126] 2020	Hybrid Features + SVM	BACH	92.2
	Hybrid Features + MLP		85.2
	Hybrid Features + RF		80.2
	Hybrid Features + XGBoost		82.7
[87] 2020	Attention Guided CNN	BACH	93.0
[99] 2020	Random Forest	BACH	91.2
	SVM		95.0
	XGBoost		42.5
	MLP		91.0
[104] 2021	MA-MIDN	BACH	93.57
[108] 2021	AhoNet (Resnet18 + ECA + MPN-COV)	BACH	85.0
[101] 2021	Inception V3 + XGBoost	BACH	87.0
[127] 2022	DSAGu-CNN	BACH	96.47
Ours	DEEP_Pachi	BACH	99.9

## Data Availability

The dataset used in this paper is public and can be obtained from these repositories: https://www.kaggle.com/ambarish/breakhis (accessed: 12 March 2022) and https://iciar2018-challenge.grand-challenge.org/Dataset/ (accessed: 12 March 2022). The TensorFlow/Keras code we used in our experiment is not yet publicly available and will be made so after the publication of the work.
